# Intrathyroidal Parathyroid Carcinoma: An Atypical Thyroid Lesion

**DOI:** 10.3389/fendo.2018.00641

**Published:** 2018-11-01

**Authors:** Noran Alharbi, Sylvia L. Asa, Marta Szybowska, Raymond H. Kim, Shereen Ezzat

**Affiliations:** ^1^Department of Internal Medicine and Endocrinology, Prince Mohammed bin Abdulaziz Hospital, Riyadh, Saudi Arabia; ^2^Department of Medicine, University Health Network, University of Toronto, Toronto, ON, Canada; ^3^Department of Pathology, University Health Network, University of Toronto, Toronto, ON, Canada; ^4^Fred A Litwin Family Centre in Genetic Medicine, University Health Network, University of Toronto, Toronto, ON, Canada

**Keywords:** thyroid nodule, parathyroid carcinoma, hypercalcemia, pathology, sorafenib

## Abstract

Parathyroid carcinoma is a rare endocrine malignancy that is typically difficult to diagnose at presentation. Here, we report a 63 year-old man who had symptomatic hypercalcemia. Investigations revealed a thyroid nodule and a lateral neck mass that was biopsied and diagnosed as “suspicious for a neuroendocrine neoplasm.” He underwent total thyroidectomy with central and left neck node dissection. Histology and immunohistochemistry revealed an intrathyroidal angioinvasive parathyroid carcinoma with lymph node metastases. The tumor showed loss of parafibromin expression; germline testing revealed no pathogenic germline variants of *CDC73*, suggesting either a cryptic germline variant or a sporadic malignancy. Multiple pulmonary nodules consistent with metastatic disease explained persistent hypercalcemia and the patient was treated with denosumab as well as Sorafenib resulting in early regression of the lung nodules. This case illustrates an unusual parathyroid carcinoma with respect to anatomic presentation and the importance of complete pathological workup in securing the diagnosis. The management of these rare malignancies is discussed.

## Background

Parathyroid carcinoma is a rare disease that must be distinguished from other types of parathyroid pathologies (1). It is a particularly difficult diagnosis to establish when the disease is detected outside of its typical anatomical sites (1). Of these, the intrathyroidal occurrence of parathyroid carcinoma can be problematic. Here, we present a case of intrathyroidal parathyroid carcinoma that demonstrates the challenges in diagnosis as well as in endocrine and oncologic control of this disease. The patient provided written informed consent for publication of this case report.

## Case presentation

A 63 year-old gentleman with known hypertension presented in October 2016 with diffuse bony aches, polyuria, polydipsia, constipation, fatigue, loss of appetite, and weight loss of 25 pounds over two months. Biochemical testing revealed normal thyroid function tests but an elevated serum calcium of 4.17 mmol/L (Normal range 2.2–2.5), phosphate 1.4 mmol/L (0.74–1.52), parathyroid hormone (PTH) 168.2 pmol/L (1.6–9.4), serum creatinine 237 umol/L (64–111), glomerular filtration rate (GFR) 24 (>60), 24 h urinary calcium 13.89 mmol/day (2.5–7.5). His past medical history was remarkable for a kidney stone 12 years earlier although he denied bony fractures of the spine and hips or renal dysfunction. Family history was non-contributory with no known history of parathyroid disease or endocrine neoplasia.

**Figure 1:**
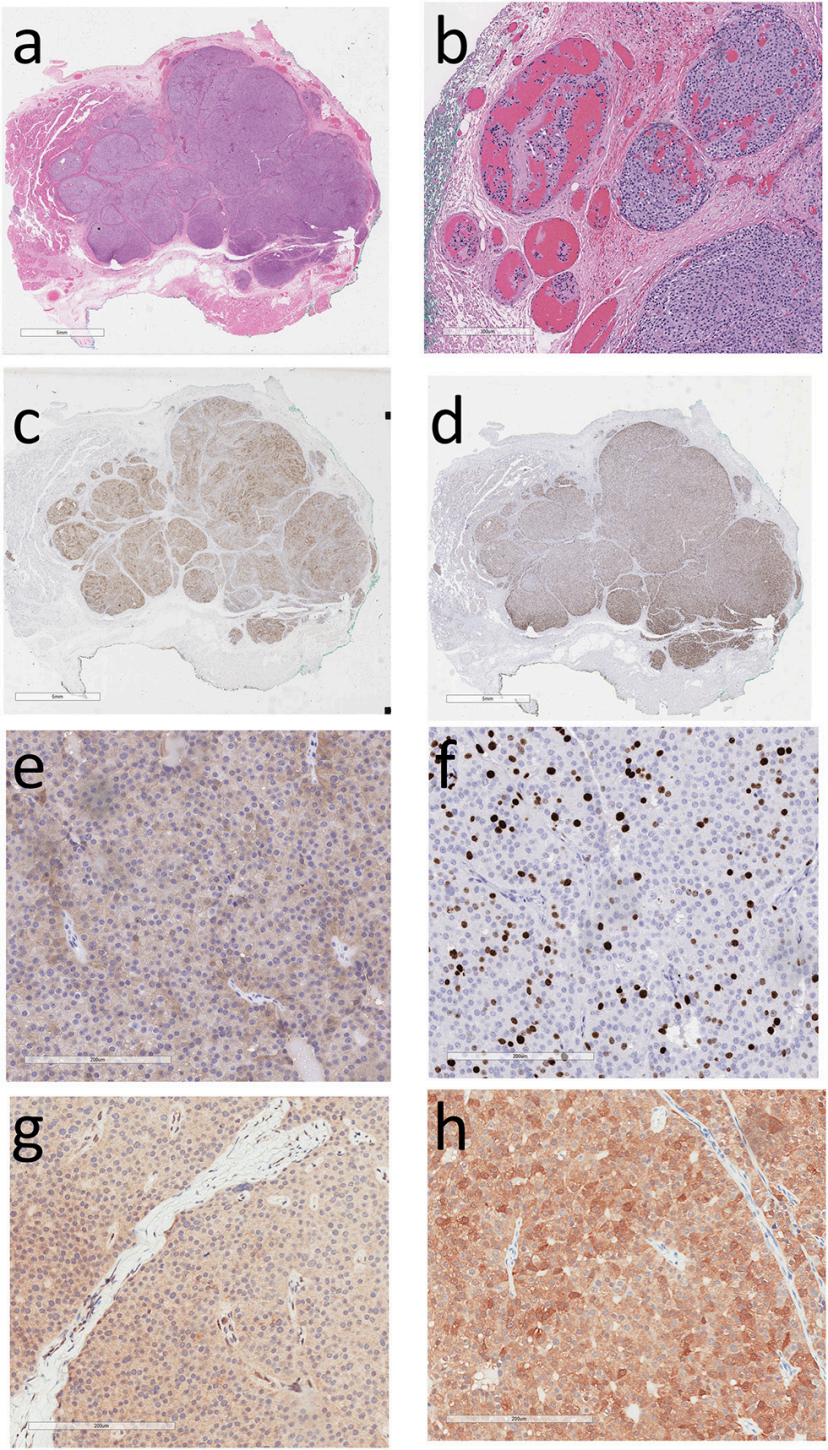
Histology and Immunoprofile of Intrathyroidal Parathyroid Carcinoma. **(a)** The tumor is infiltrative within the thyroid. **(b)** There is vascular invasion characterized by intravascular tumor cells admixed with thrombus. **(c)** The tumor cells exhibit cytoplasmic reactivity for parathyroid hormone and **(d)** nuclear staining for GATA-3, confirming parathyroid differentiation. **(e)** Cytoplasmic reactivity for Galectin-3 is a feature suggesting parathyroid carcinoma. **(f)** The Ki67 labeling index is approximately 20%. **(g)** The tumor cells show loss of nuclear parafibromin reactivity and **(h)** there is diffuse staining for PGP 9.5.

His treating physicians noted a left thyroid nodule and a left neck node which was biopsied; cytologic examination revealed features suspicious for a neuroendocrine neoplasm. He underwent a total thyroidectomy with central and left neck node dissection. Review of the pathology in the thyroidectomy specimen revealed that the index thyroid mass was indeed an infiltrative intrathyroidal neuroendocrine tumor (Figure 1a) that measured 2.7 cm and had multiple foci of vascular invasion characterized by intravascular tumor cells admixed with thrombus (Figure 1b). There was single cell tumor necrosis. Mitoses, including atypical mitoses, were conspicuous and a phospho-histone 3 (pHH3)-assisted mitotic count identified 26 mitotic figures per 50 high power fields. The tumor was positive for keratins using the CAM5.2 and CK7 antibodies, CD56, chromogranin, PTH (Figure 1c), and GATA-3 (Figure 1d), confirming that it was indeed a parathyroid neoplasm; it was negative for CK20, S100, CD5, Pax-8, TTF-1, thyroglobulin, CEA, and calcitonin. The case was then evaluated to confirm biomarkers of malignancy in parathyroid tumors. It was positive for Galectin-3 (Figure 1e); The Ki67 labeling index was 19.6% in an automated count of 1158 cells (Leica Biosystems; Figure 1f), there was reduced expression of BCL-2 and focal upregulation of cyclin D1 and p53. There was no loss of RB but marked reduction of p27. Staining for menin was technically unsatisfactory. There was loss of nuclear parafibromin reactivity (Figure 1g) and PGP 9.5 was positive (Figure 1h).

The painted margin of resection of the thyroidectomy was negative for malignancy, however, metastatic parathyroid carcinoma was identified in one left perithyroidal lymph node included in the total thyroidectomy and central neck dissection specimen and in one of 38 lymph nodes from the left neck dissection specimen. A left inferior parathyroid gland was biopsied and had normal morphology.

Elsewhere in the thyroid there was an incidental 0.05 cm classical variant papillary microcarcinoma.

**Figure 2:**
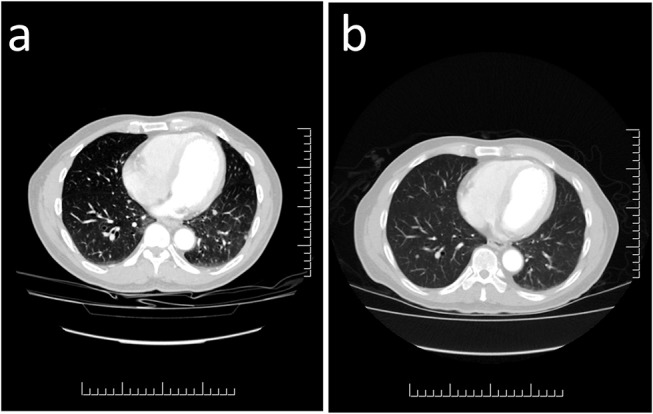
Computed Tomography Imaging of Metastatic Parathyroid Carcinoma. **(a)** Multiple scattered lesions throughout both lung fields are consistent with metastatic parathyroid carcinoma. **(b)** Repeat imaging 3 months following treatment with the tyrosine kinase inhibitor sorafenib demonstrates reduction in the size of several of the pulmonary nodules which are now less prominent.

Post-operatively, he noted a marked improvement in his symptoms with reduction of corrected serum calcium to 2.58 mmol/L and PTH to 12.9 pmol/L. However a few months later, his PTH level remained elevated precipitating a referral to our institution. Our investigations yielded the following findings: serum PTH of 27.7 pmol/L (1.3–7.6), corrected calcium 2.84 mmol/L (2.32–2.62), phosphate 0.55 mmol/L (0.8–1.4), 25(OH) vitamin D3 84 nmol/L (25–200), ALP 43 U/L (40–150), creatinine 107 umol/L (64–110), and calculated GFR 64 ml/min/1.73 m^2^ (> 60). CT imaging identified no visible disease in the neck but multiple pulmonary nodules consistent with metastatic disease were noted (Figure 2a). An abdominal ultrasound showed multiple renal cysts. Nuclear octreotide scintigraphy was completely negative.

Based on the clinical findings genetic testing was performed with sequencing and deletion/duplication analysis of *CDC73*. No pathogenic variants were detected. He was started on denosumab 120 mg subcutaneously monthly to help control his hypercalcemia. To control his structural metastatic disease, targeted therapy with sorafenib 400 mg orally twice daily was started. Three months later, CT imaging showed early regression of the lung nodules (Figure 2b).

## Discussion

Parathyroid glands can be located in various ectopic locations in the neck (1). The superior parathyroids are usually located close to the posterior thyroid near the cricothyroid junction; they may be in perithyroidal fascia or completely intrathyroidal. They may also be retropharyngeal or retroesophageal. The inferior glands may be associated with the thymus anywhere from near the inferior pole of the thyroid to lower in the mediastinum or higher, even associated with the hyoid bone (2). We report a parathyroid carcinoma that was completely intrathyroidal, consistent with neoplasia arising in an intrathyroidal superior parathyroid gland. The patient's ipsilateral inferior parathyroid gland was identified and biopsied and proved to be normal on histology.

Parathyroid carcinoma comprises ~1% of all cases of primary hyperparathyroidism (PHPT) (1). Risk factors include long-standing secondary hyperparathyroidism or previous history of head and neck irradiation. Parathyroid cancer has been associated with a rare autosomal dominant inherited disorder known as hyperparathyroidism–jaw tumor syndrome (HPT-JT) due to germline mutations in the *CDC73* gene that encodes parafibromin (1). Less frequently, it is associated with familial isolated hyperparathyroidism and multiple endocrine neoplasia type 1 and type 2A (1).

The classic symptoms of PHPT include many complications such as kidney stones, bony loss, abdominal pain, and neuropsychiatric complaints. As opposed to the more benign form of the disease, patients with parathyroid cancer are usually severely symptomatic at the time of presentation with severe hypercalcemia. Indeed, severe nephrolithiasis, nephrocalcinosis, and impaired renal function with bone loss is noted in nearly 80% of affected patients (3). The latter may include osteitis fibrosa cystica, diffuse osteopenia, or even pathologic fractures. Other constitutional symptoms associated with PHPT such as fatigue, loss of concentration, bony pain, polydipsia, polyuria, peptic ulcerations, pancreatitis, and depression may also be noted. Other manifestations of severe hypercalcemia including acute pancreatitis, shortened QT interval, drowsiness, and diminished level of consciousness, which if left untreated, can be fatal.

In addition to the stigmata of hyperparathyroidism, a palpable neck mass, such as in our case, can be present in 30–75% of patients with parathyroid carcinoma (3). It should be emphasized that this latter finding is quite rare in benign forms of parathyroid disease. More recent series, however, indicate that a palpable mass may be less common. Kleinpeter et al noted a palpable mass in only 22% of their patients with parathyroid carcinomas (4). Hoarseness, resulting from recurrent laryngeal nerve palsy, and palpably enlarged lymph nodes can also provide a clue to the presence of a parathyroid carcinoma.

The occurrence of an intrathyroidal parathyroid carcinoma is rare but not unknown (5–17). The complexity of the diagnosis in the face of a thyroid nodule rests on the clinical suspicion of severe hyperparathyroidism with an infiltrative thyroid mass. However, the more common scenario is a parathyroid lesion associated with an unrelated thyroid mass. In this case, the lack of resolution of biochemical abnormalities after surgery prompted pathology review to ensure that the culprit parathyroid was indeed resected, since it was possible that the thyroid lesion was unrelated and the abnormal parathyroid might have been left behind in a different ectopic location.

The diagnosis of malignancy in a parathyroid neoplasm should be suspected in patients with PHPT if at the time of neck exploration, the mass is large, white or gray, and adherent to adjacent structures. This is in contradistinction to benign parathyroid neoplasms, which tend to be soft, flat, and more red-brown in color and easy to dissect surgically. Surgical pathology remains the cornerstone of diagnosis. Carcinomas are typically larger, tend to be more firm, irregularly-shaped, and have a whitish-gray color; they are often adherent or invasive into surrounding neck structures. However, benign parathyroid lesions can exhibit worrisome features such as fibrosis and degeneration if they have been subjected to pre-operative biopsy (18, 19). This is a common finding especially in patients who present with a thyroid mass, as in our case. The diagnosis of malignancy therefore requires identification of unequivocal angioinvasion and can be assisted by the assessment of biomarkers that are features of parathyroid malignancy (20). Our case showed loss of BCL-2, overexpression of galectin-3, reduction of p27, and a high Ki67 labeling index that all support the diagnosis of malignancy. Negative staining for parafibromin and positive staining for PGP9.5 is an abnormal pattern which is very suggestive of *CDC73* mutation/inactivation.

Currently there are no formal clinical diagnostic criteria for HPT-JT, and the diagnosis relies on the detection of a pathogenic variant in *CDC73*. Sequencing (21) and copy number analysis (22) of *CDC73* is highly sensitive for HPT-JT, but not all variants in *CDC73* have been characterized (22). Our patient did not have an identifiable germline pathogenic variant in the *CDC73* gene, suggesting that an undetectable variant germline variant may be present or the tumor may have had a somatically acquired mutation or epigenetic silencing of this gene. Renal manifestations including renal cysts can be a finding in HPT-JT (23). Despite our patient's negative genetic testing results, the findings of absent parafibromin expression in his tumor and the presence of renal cysts were highly suggestive of HPT-JT, and family members were also recommended to have HPT-JT surveillance.

Surgery is currently the only effective form of treatment for parathyroid carcinoma (1). Complete resection avoiding capsular disruption is recommended (1). Outcome studies have shown that complete resection with free margins is a feature of tumors that do well (24). Although radiotherapy has been reported to have beneficial effects (24, 25), it is not recommended as a routine ancillary tool, rather it is reserved for palliation (1). Conventional chemotherapy does not effectively correct hypercalcemia nor influence overall outcomes from the disease (26). A large number of agents including cyclophosphamide, 5-fluorouracil and decarbazine have been tested as single or combination therapies without appreciable effects. Partial responses with a marked drop in serum calcium lasting from weeks to several months have been observed in some individual cases; however, in general the results are largely disappointing. Denosumab, a monoclonal antibody against RANKL, inhibits osteoclast maturation, function, and survival and is useful to treat hypercalcemia of malignancy, including in patients with parathyroid carcinoma (27).

Recurrence of tumors can be monitored by serum calcium and parathyroid hormone levels. However, in view of the potential for loss of differentiation with cancer progression, serum PTH alone may not be fully revealing and long-term imaging surveillance is often required for patients with parathyroid carcinoma. Most recurrences occur within 2–3 years from the time of initial presentation, but prolonged disease-free interval of as long as 23 years has also been reported in some cases (28).

## Author contributions

NA, SA, RK, and SE: case review; NA, SA, and SE: writing; NA and SA: image preparation; RK, MS, and SE: review of manuscript.

### Conflict of interest statement

The authors declare that the research was conducted in the absence of any commercial or financial relationships that could be construed as a potential conflict of interest.

## References

[1] Wilhelm SM Wang TS Ruan DT Lee JA Asa SL Duh QY. The American association of endocrine surgeons guidelines for definitive management of primary hyperparathyroidism. JAMA Surg. (2016) 151:959–68. 10.1001/jamasurg.2016.2310 27532368

[2] Asa SL Mete O Parathyroids. In: Mills SE, editor. Histology for Pathologists, Philadelphia, PA: Wolters Kluwer (2018).

[3] Fang SH Lal G. Parathyroid cancer. Endocr Pract (2011) 17(Suppl. 1):36–43. 10.4158/EP10310.RA 21454239

[4] Kleinpeter KP Lovato JF Clark PB Wooldridge T Norman ES Bergman S. Is parathyroid carcinoma indeed a lethal disease? Ann Surg Oncol. (2005) 12:260–6. 10.1245/ASO.2005.03.036 15827819

[5] Crescenzo DG Shabahang M Garvin D Evans SR. Intrathyroidal parathyroid cancer presenting as a left neck mass. Thyroid (1998) 8:597–9. 10.1089/thy.1998.8.597 9709913

[6] Kirstein LJ Ghosh BC. Intrathyroid parathyroid carcinoma. J Surg Oncol. (2001) 77:136–8. 10.1002/jso.1084 11398168

[7] Schmidt JL Perry RC Philippsen LP Wu HH. Intrathyroidal parathyroid carcinoma presenting with only hypercalcemia. Otolaryngol Head Neck Surg. (2002) 127:352–3. 10.1067/mhn.2002.128553 12402018

[8] Hussein WI El-Maghraby TA Al-Sanea O. Hyperfunctioning intrathyroidal parathyroid carcinoma. Saudi Med J. (2006) 27:1226–9. 16883457

[9] Foppiani L Del MP Sartini G Arlandini A Quilici P Bandelloni R. Intrathyroidal parathyroid carcinoma as cause of hypercalcemia and pitfall of localization techniques: clinical and biologic features. Endocr Pract. (2007) 13:176–81. 10.4158/EP.13.2.176 17490933

[10] Temmim L Sinowatz F Hussein WI Al-Sanea O El-Khodary H. Intrathyroidal parathyroid carcinoma: a case report with clinical and histological findings. Diagn Pathol. (2008) 3:46. 10.1186/1746-1596-3-46 19032735 PMC2627811

[11] Herrera-Hernandez AA Aranda-Valderrama P Diaz-Perez JA Herrera LP. Intrathyroidal parathyroid carcinoma in a pediatric patient. Pediatr Surg Int. (2011) 27:1361–5. 10.1007/s00383-011-2904-6 21519840

[12] Quartey B Shriver C Russell D. Intrathyroidal parathyroid carcinoma presenting as asymptomatic high normal serum calcium and slightly elevated intact parathyroid hormone: a case report and review of literature. World J Oncol. (2011) 2:138–42. 10.4021/wjon311w 29147238 PMC5649667

[13] Kruljac I Pavic I Matesa N Mirosevic G Maric A Becejac B. Intrathyroid parathyroid carcinoma with intrathyroidal metastasis to the contralateral lobe: source of diagnostic and treatment pitfalls. Jpn J Clin Oncol. (2011) 41:1142–6. 10.1093/jjco/hyr094 21742652

[14] Vila DL Winter WE Vaysberg M Moran CA Al-Quran SZ. Intrathyroidal parathyroid carcinoma: report of an unusual case and review of the literature. Case Rep Pathol. (2013) 2013:198643. 10.1155/2013/198643 23936709 PMC3725913

[15] Lee KM Kim EJ Choi WS Park WS Kim SW. Intrathyroidal parathyroid carcinoma mimicking a thyroid nodule in a MEN type 1 patient. J Clin Ultrasound (2014) 42:212–4. 10.1002/jcu.22090 24037737

[16] Tejera Hernandez AA Gutierrez Giner MI Vega B V Fernandez San MD Hernandez Hernandez JR. Intrathyroidal parathyroid carcinoma. a case report and review of literature. Endocrinol Nutr. (2016) 63:46–8. 10.1016/j.endonu.2015.09.004 26588997

[17] Wong YP Sharifah NA Tan GC Gill AJ Ali SZ. Intrathyroidal oxyphilic parathyroid carcinoma: a potential diagnostic caveat in cytology? Diagn Cytopathol. (2016) 44:688–92. 10.1002/dc.23493 27229757

[18] Alwaheeb S Rambaldini G Boerner S Coire C Fiser J Asa SL. Worrisome histologic alterations following fine-needle aspiration of the parathyroid. J Clin Pathol. (2006) 59:1094–6. 10.1136/jcp.2005.029017 17021134 PMC1861746

[19] Kim J Horowitz G Hong M Orsini M Asa SL Higgins K. The dangers of parathyroid biopsy. J Otolaryngol Head Neck Surg. (2017) 46:4. 10.1186/s40463-016-0178-7 28061891 PMC5219743

[20] Erovic BM Harris L Jamali M Goldstein DP Irish JC Asa SL. Biomarkers of parathyroid carcinoma. Endocr Pathol. (2012) 23:221–31. 10.1007/s12022-012-9222-y 23001705

[21] van der Tuin K Tops CMJ Adank MA Cobben JM Hamdy NAT Jongmans MC. CDC73-related disorders: clinical manifestations and case detection in primary hyperparathyroidism. J Clin Endocrinol Metab. (2017) 102:4534–40. 10.1210/jc.2017-01249 29040582

[22] Bricaire L Odou MF Cardot-Bauters C Delemer B North MO Salenave S. Frequent large germline HRPT2 deletions in a French National cohort of patients with primary hyperparathyroidism. J Clin Endocrinol Metab. (2013) 98:E403–8. 10.1210/jc.2012-2789 23293331

[23] Tan MH Teh BT. Renal neoplasia in the hyperparathyroidism-jaw tumor syndrome. Curr Mol Med. (2004) 4:895–7. 10.2174/1566524043359719 15579037

[24] Erovic BM Goldstein DP Kim D Mete O Brierley J Tsang R. Parathyroid cancer: outcome analysis of 16 patients treated at the princess margaret hospital. Head Neck (2012) 35:35–9. 10.1002/hed.22908 22290780

[25] Rasmuson T Kristoffersson A Boquist L. Positive effect of radiotherapy and surgery on hormonally active pulmonary metastases of primary parathyroid carcinoma. Eur J Endocrinol. (2000) 143:749–54. 10.1530/eje.0.1430749 11124857

[26] Kassahun WT Jonas S. Focus on parathyroid carcinoma. Int J Surg. (2011) 9:13–9. 10.1016/j.ijsu.2010.09.003 20887820

[27] Thosani S Hu MI. Denosumab: a new agent in the management of hypercalcemia of malignancy. Future Oncol. (2015) 11:2865–71. 10.2217/fon.15.232 26403973 PMC4976858

[28] Al-Fadhli M Doi SA Muttikkal T Al-Sumait B. Severe hyperparathyroidism versus parathyroid carcinoma: a clinical dilemma. Sultan Qaboos Univ Med J. (2010) 10:94–100. 21509088 PMC3074659

